# Clinical trial protocol: PRednisolone in early diffuse cutaneous Systemic Sclerosis (PRedSS)

**DOI:** 10.1177/2397198320957552

**Published:** 2020-09-17

**Authors:** Ariane L Herrick, Deborah J Griffiths-Jones, W David Ryder, Justin C Mason, Christopher P Denton

**Affiliations:** 1 Division of Musculoskeletal and Dermatological Sciences, Salford Royal NHS Foundation Trust, Manchester Academic Health Science Centre, The University of Manchester, Manchester, UK; 2 Manchester Clinical Trials Unit, Jean McFarlane Building, The University of Manchester, Manchester, UK; 3 National Heart and Lung Institute, Imperial College London, Hammersmith Hospital, London, UK; 4 Centre for Rheumatology and Connective Tissue Diseases, University College London Division of Medicine, Royal Free Campus, London, UK

**Keywords:** Diffuse cutaneous systemic sclerosis, prednisolone, skin score, disability, pain

## Abstract

**Background:**

Many of the painful, disabling features of early diffuse cutaneous systemic sclerosis have an inflammatory component and are potentially treatable with corticosteroid therapy. These features include painful and itchy skin, fatigue and musculoskeletal involvement. Yet many clinicians are understandably reluctant to prescribe corticosteroids because of the concern that these are a risk factor for scleroderma renal crisis. The aim of PRedSS (PRednisolone in early diffuse cutaneous Systemic Sclerosis) is to evaluate the efficacy and safety of moderate dose prednisolone in patients with early diffuse cutaneous systemic sclerosis, specifically whether moderate dose prednisolone is (a) effective in terms of reducing pain and disability, and improving skin score and (b) safe, with particular reference to renal function.

**Methods:**

PRedSS is a Phase II, multicentre, double-blind randomised controlled trial which aims to recruit 72 patients with early diffuse cutaneous systemic sclerosis. Patients are randomised to receive either prednisolone (dosage approximately 0.3 mg/kg) or placebo therapy for 6 months. The two co-primary outcome measures are the difference in mean Health Assessment Questionnaire Disability Index at 3 months and the difference in modified Rodnan skin score at 3 months. Secondary outcome measures include patient reported outcome measures of itch, hand function, anxiety and depression, and helplessness.

**Results:**

Recruitment commenced in December 2017 and after a slow start (due to delays in opening centres) 25 patients have now been recruited.

**Conclusion:**

PRedSS should help to answer the question as to whether clinicians should or should not prescribe prednisolone in early diffuse cutaneous systemic sclerosis.

## Introduction

Diffuse cutaneous systemic sclerosis (dcSSc) is a potentially devastating illness with a very major impact on quality of life as well as on survival. Affected patients typically experience rapid progression of skin thickening, commencing distally but going on to involve proximal limb and/or trunk, often with early involvement of internal organs. Early dcSSc is characterised by pain, stiffness, disability, disfigurement, fatigue, intractable itch and (often) a feeling of helplessness. Currently, there is no cure. The major impact on quality of life in patients with early dcSSc is in the context of a 10-year survival rate of little more than 50%.^1^ Risk factors for death include extensive skin involvement, early internal organ involvement (lung, heart and kidney) and anti-topoisomerase (anti-Scl70) antibodies.^2,3^ Understandably, clinicians caring for patients with early dcSSc have therefore tended to focus on early identification and treatment of internal organ involvement, and on immunosuppressive therapy,^4,5^ on the basis that there is evidence of immune activation with inflammation early in the disease course.^6,7^ Although randomised controlled trials (RCTs)^8,9^ have shown that haemopoetic stem cell transplantation (HSCT) confers survival advantage, these trials were in highly selected patients, and HSCT tends to be reserved for those patients with the poorest prognosis.

Focusing on the internal organ involvement of early dcSSc risks ignoring the aspects of disease which have the most impact upon patients’ day-to-day lives, namely, painful and itchy skin, fatigue and loss of function due to a combination of early contractures (especially of the fingers) and musculoskeletal involvement. This musculoskeletal involvement includes tendinitis, myositis and/or joint inflammation which may be unrecognised. Many of these early features which severely impact on quality of life have an inflammatory component and are potentially treatable with steroid therapy. However, corticosteroids are much less frequently prescribed in patients with systemic sclerosis (SSc) than in patients with other connective tissue diseases. This is because (a) SSc is not generally considered primarily an inflammatory disease (instead being characterised by fibrosis and ischaemia, although as stated above, there is a degree of inflammation in early diffuse disease) and (b) corticosteroids are a risk factor for renal crisis,^10–12^ which is most likely to occur in that subset of patients with early diffuse cutaneous disease. Patients who are anti-RNA polymerase III antibody positive are at particular risk of renal crisis.^13,14^

Hence, there is a clinical dilemma: should corticosteroids be prescribed or not?^7,15^ Anecdotally, many clinicians with an interest in SSc prescribe low dose corticosteroids (e.g. prednisolone 10 mg/day or less) and report that patients are symptomatically improved. However, other clinicians will not prescribe corticosteroids because of understandable concerns about adverse effects (hypertension, renal crisis), although open-label studies in patients with early dcSSc including low to medium dose corticosteroids did not report renal crises^16–18^ and several other studies in patients with SSc in which treatment regimens have included steroid therapy have not reported adverse renal outcomes (reviewed in 15). The difference in opinion as to whether steroids should or should not be prescribed in early dcSSc was recently well illustrated by experience from the European Scleroderma Observational Study (ESOS)^19^: of 326 patients recruited, 141 (44%) reported current or previous corticosteroid use. A meta-analysis published in 2014 also reported a high prevalence of corticosteroid use in patients with dcSSc.^20^

**Figure 1.**
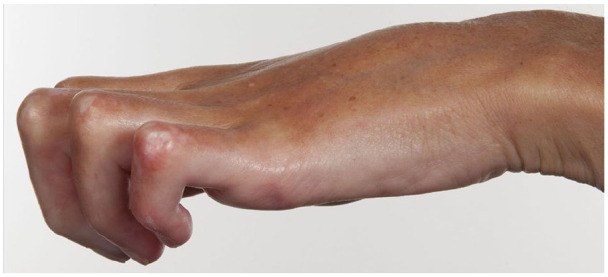
Finger flexion contractures in a patient with established dcSSc.

Against this background, our overall hypothesis is that moderate dose prednisolone confers symptomatic and functional benefit in patients with early dcSSc and that the benefit outweighs any side effects. This hypothesis relates specifically to patients with early diffuse (as opposed to limited) cutaneous SSc. The main benefits are expected through anti-inflammatory effects in the skin and in the musculoskeletal system, thus preventing late and irreversible complications including finger contractures (Figure 1). Therefore, the overall aim of ‘PRednisolone in early diffuse cutaneous Systemic Sclerosis’ (PRedSS) is to evaluate the efficacy and safety of moderate dose prednisolone in early dcSSc. Specific objectives are to evaluate whether

1. Moderate dose prednisolone is effective in patients with early dcSSc in terms of reducing pain and disability and improving skin score.
2. Moderate dose prednisolone is a safe therapy in patients with early dcSSc (with particular reference to renal function).

## Methods

### Study design

PRedSS (ClinicalTrials.gov Identifier: NCT03708718) is a Phase II, multicentre, double-blind RCT which will evaluate the efficacy and safety of moderate dose prednisolone in 72 patients with early dcSSc over a 6-month period, with the co-primary endpoints (see below) being assessed at 3 months. The decision to have the primary endpoint at 3 (as opposed to 6) months was taken to maximise patient retention in the study up until the primary endpoint. The study was approved by the North West – Greater Manchester South Research Ethics Committee on the 28 June 2017: all patients sign informed consent.

#### Randomisation

**Figure 2.**
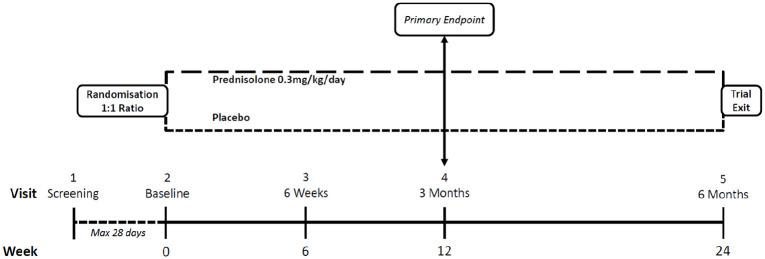
Study design.

Eligible patients are randomised 1:1 to receive either enteric-coated prednisolone or matching placebo capsules (1 active capsule = 5-mg prednisolone) (Figure 2). Randomisation is stratified according to whether the patient is positive for anti-topoisomerase antibody. Although ideally randomisation would have been stratified for anti-RNA polymerase III positivity, this was deemed too logistically difficult because not all participating centres have rapid access to testing for anti-RNA polymerase III. Because anti-topoisomerase antibody and anti-RNA polymerase III are almost invariably mutually exclusive, it is likely that this chosen approach will result in similar numbers of patients who are anti-RNA polymerase III antibody positive being randomised to each of the two treatment groups.

#### Treatment

The dosage schedules have been designed so that patients receive approximately 0.3 mg/kg of prednisolone or less: weight <50 kg = 10 mg; ⩾50 kg but <60 kg = 15 mg; ⩾60 kg but <80 kg = 20 mg, ⩾80 kg but <100 kg = 25 mg; ⩾100 kg = 30 mg. These doses were chosen because they are thought to be high enough to be associated with substantial anti-inflammatory effects, but at the same time not so high as to deter clinicians concerned about the renal side effects thought to be associated with high dose. If a patient experiences adverse effects deemed in the opinion of the investigator to be likely related to trial treatment and to warrant reducing the dose of prednisolone (or placebo equivalent), then the dose may be reduced at the clinician’s discretion, and the reason recorded. This trial treatment is additive to (and not a substitute for) any other therapies which might be prescribed. For example, as described below under inclusion and exclusion criteria, a patient on immunosuppressant therapy may be entered into the trial. This reflects the ‘real-world’ situation post-RCT if prednisolone is found to be effective: prednisolone will be prescribed in addition to other therapies. A proton pump inhibitor, and a calcium and vitamin D supplement, are co-prescribed with the trial treatment on the basis that there is a 50% chance that this is prednisolone which may cause upper gastro-intestinal side effects and which is a risk factor for osteoporosis.

#### Visit schedule and study exit

Patients attend on five occasions (Figure 2): a screening visit (up to 28 days before the baseline visit), baseline, 6 weeks, 3 months and 6 months. At the 6-month (final) visit, the treatment code is broken, because corticosteroid therapy cannot be stopped suddenly and it is therefore important that both clinician and patient know whether the patient is on prednisolone therapy. For those patients randomised to prednisolone, the clinician then makes a decision as to whether or not to continue the current dose, whether to reduce the dose or whether to gradually taper the dose with a view to discontinuing.

The trial will complete when the last patient has completed the 6-month visit. Any patients discontinuing treatment prior to their 6-month visit are asked to attend all visits and complete all assessments as previously intended.

### Patients

Inclusion and exclusion criteria are listed in Table 1.

**Table 1.** Inclusion and exclusion criteria.

Inclusion criteria	
a.	Aged 18 years or more
b.	DcSSc (with skin involvement extending to proximal limb and/or trunk)
c.	Skin involvement of less than 3 years
Exclusion criteria	
a.	Previous renal crisis or significant renal impairment (estimated glomerular filtration rate (GFR) < 40 mL/min)
b.	Currently on corticosteroid therapy, or previous corticosteroid therapy (with the exception of inhaled or topical steroids) within the last 4 weeks.
c.	Currently on an immunosuppressant (e.g. mycophenolate mofetil, methotrexate) or biologic therapy the dose of which has changed in the previous 4 weeks, or is likely to change during the first 3 months of trial treatment. Patients on immunosuppressant therapies should ideally remain on the same dose throughout the study period, although it is recognised that this may not be possible.
d.	Patients with significant uncontrolled Stage 1 hypertension (clinic BP >140/90 mmHg). Patients with previous hypertension which is controlled for at least 4 weeks are considered eligible.
e.	Any condition which in the opinion of the attending clinician would make corticosteroid therapy unwise (e.g. chronic infection, unstable diabetes, uncontrolled blood pressure).
f.	Patients with major myositis or inflammatory arthritis. Patients with low-level myositis or inflammatory arthritis are eligible for inclusion (e.g. in the case of myositis, a creatine kinase less than 4 times the upper limit of normal or myositis only demonstrable on magnetic resonance imaging).
g.	Female patients who are pregnant at time of screening.
h.	Female patients who are breastfeeding.
i.	Patients with significant inflammatory bowel disease as judged by the investigator.
j.	Patients currently participating in another randomised controlled trial.
k.	Patients who do not fully understand the importance of not suddenly stopping taking the study medication.

### Outcome measures

Primary and secondary efficacy outcome measures, and safety measures, are shown in Table 2.

**Table 2.** Efficacy outcome measures and safety measures.

Primary outcomes	Assessed at 3 months (Visit 4)	Functional ability as measured by the Health Assessment Questionnaire Disability Index (HAQ-DI)
		Modified Rodnan skin score (mRSS)
Secondary outcomes	Assessed at: 6 weeks (Visit 3) 3 months (Visit 4) 6 months (Visit 5)	HAQ-DI (6 weeks and 6 months)
		mRSS (6 weeks and 6 months)
		Perceived pain as measured by the HAQ Visual Analogue Scale (HAQ VAS)
		Multisystem effects of SSc as measured by the Scleroderma Health Assessment Questionnaire Visual Analogue Scales (SHAQ VAS)
		Functional ability as measured by the 11-point Scleroderma Functional Index (to complement the HAQ-DI)
		Scleroderma Skin Patient Reported Outcome (SSPRO)
		Pruritus as measured by the 5-D Itch Questionnaire
		Hand disability as measured by the Cochin Hand Function Scale (CHFS)
		Fatigue as measured by the Functional Assessment of Chronic Illness Therapy (FACIT) questionnaire
		Anxiety and depression as measured by the Hospital Anxiety and Depression Scale (HADS)
		Helplessness as measured by the 5-Item helplessness subscale of the Rheumatology Attitudes Index (RAI)
		Health status, mental and physical aspects, as measured by the Short Form (36) Health Survey v2^®^ (SF-36v2^®^)
		Health utility as measured by the EuroQoL (EQ-5D-3L)
		Patient and Physician Global Assessments
		Digital ulcer count
		Tendon friction rubs
		Joint count
Safety measures	Assessed at: 6 weeks (Visit 3) 3 months (Visit 4) 6 months (Visit 5)	Blood pressure
		Renal function as assessed by: Serum creatinine Estimated Glomerular Filtration Rate Urinary protein/creatinine ratio
		Full blood count
		Blood glucose

#### Efficacy

There are two co-primary outcome measures: the difference in mean Health Assessment Questionnaire Disability Index (HAQ-DI) at 3 months and the difference in modified Rodnan skin score (mRSS) at 3 months (compared to baseline). The HAQ-DI is a self-administered questionnaire which has been widely applied in studies of early dcSSc.^21^ It includes a visual analogue scale (VAS) for pain. It captures many of the clinical features of early dcSSc expected to be improved by corticosteroids. The mRSS^22,23^ is often the primary outcome measure in trials of early dcSSc^24–26^ because it reflects the overall disease process and is a predictor of outcome.^27^ Despite the short study duration (primary endpoint at 3 months), having the mRSS as a co-primary outcome measure will allow comparisons between PRedSS and other studies of early diffuse cutaneous disease. Both HAQ-DI and mRSS are validated in SSc as per OMERACT (Outcome Measures in Rheumatology) criteria.^28^

Secondary endpoints, most of which are self-administered questionnaires, include the following:

a. The different VAS of the ‘Scleroderma HAQ’ (SHAQ)(21): in addition to pain, these relate to Raynaud’s phenomenon, digital ulceration, gastrointestinal and lung manifestations, and overall disease.
b. The 11-point Scleroderma Functional Index,^29^ which is a SSc-specific index of functional ability, measuring mainly upper limb function.
c. The Scleroderma Skin Patient Reported Outcome (SSPRO).^30^ This was added 10 months after recruitment had commenced, because it only became available after the study had started.
d. The 5-D Itch scale.^31^ Itch adversely affects quality of life in patients with SSc,^32^ and patients with dcSSc attending the user group meeting which informed PRedSS considered itch as one of the most troublesome symptoms.
e. The Cochin Hand Function Scale,^33^ which has been used extensively in studies of SSc and which associates with the mRSS.^34^
f. The Functional Assessment of Chronic Illness Therapy (FACIT) questionnaire. This quantifies fatigue^35,36^ which is a significant symptom in patients with SSc^37^ including in patients with early dcSSc.^34^
g. The Hospital Anxiety and Depression (HADS)^38^ Questionnaire, which quantifies anxiety and depression and which has been widely applied in patients with SSc.^39,40^
h. A helplessness questionnaire. The five Rheumatology Attitudes Index (RAI) helplessness items are extracted from a larger helplessness scale, the RAI.^41,42^
i. The Short-form 36 (SF-36v2^®^)^43^ and EuroQol-5D (EQ-5D)^44^ questionnaires, which measure health-related quality of life (HRQOL).
j. Patient global assessment.

Non-patient reported outcome measures are as follows:

k. Physician global assessment;
l. Examination findings: tender and swollen joint count,^45^ number of tendon friction rubs and number of digital (finger) ulcers.

We were concerned at the potential ‘questionnaire burden’ of the study, and therefore patient representatives were asked to complete the questionnaires before the final decision as to which ones to include was made. The opinion was that all should be included, because the patients felt it was important to gain as much information as possible from the study. Once finalised, the questionnaire pack was sent to a small sample of patients to assess average time taken. The questionnaires take approximately 15–25 min to complete, which patients thought was feasible. The Composite Response Index in Systemic Sclerosis (CRISS) index^46^ is not being included as a secondary outcome because it includes lung function, which is not being assessed in this study.

#### Safety

The main safety endpoints are changes in blood pressure and in renal function (serum creatinine, estimated glomerular filtration rate, urinary protein/creatinine ratio) and any serious adverse events including infections. Patients are given the option of a personal blood pressure monitor and asked to check and record their blood pressure at home at least twice weekly, with advice to report immediately any new symptoms (e.g. new breathlessness or headache) which could denote a sudden rise in blood pressure. This voluntary self-assessment is not related to any of the outcome measures. A full blood count and blood glucose are also checked at each visit.

### Statistical aspects

#### Sample size calculation

This was based on HAQ-DI and mRSS as co-primary endpoints and employed a reduced 2-tail significance level of 2.5% for each. Sample size was first calculated for a two-group t-test of mean values at 3 months and then adjusted for the planned more efficient analysis of covariance (ANCOVA) analysis via a multiplier (1 − r^2^) where r is the assumed correlation between baseline and 3-month values.^47^ A standard deviation of the HAQ-DI of 0.9 was used (based on previous studies^8,48^ and data from the Salford Royal Hospital SSc database). The correlation between HAQ-DI scores at baseline and at 1-year follow-up (calculated from the clinical database) was 0.6. On the basis that we want to see a large difference in the group means at 3 months in order to proceed with prednisolone as intervention for early dcSSc, we selected a minimum clinically relevant difference of 0.6 points on the HAQ-DI. This corresponds to ‘marked improvement’ in the study of Khanna et al.^49^ which compared change in the HAQ-DI to clinician assessment. An overall sample size of 60 patients (30 per arm) then gives 82% power to detect a 0.6 difference at a 2.5% significance level, assuming analysis is performed by an ANCOVA adjusting for baseline scores. An analogous calculation was performed for mRSS, again informed by Autologous Stem cell Transplantation International Scleroderma (ASTIS)^8^ and the SSc clinical database. Assuming a standard deviation of 8.2, together with a correlation between baseline and follow-up scores of 0.9, 60 patients would then provide 99% power to detect a difference of 5.5 points on the mRSS at a 2.5% significance level. Acknowledging that this estimate of the correlation is very high and may not be repeated in the trial, we repeated the calculation assuming a more modest correlation of 0.6. Sixty patients would then also provide 82% power to detect a difference of 5.5 points on the mRSS at a 2.5% significance level. We anticipate having to recruit approximately 12 more patients to account for attrition.

#### Statistical analysis

Primary analyses will be conducted on an intention-to-treat basis; withdrawn patients will continue to complete visits and their measurements will be included in the analysis. The two co-primary outcome measures (HAQ-DI and mRSS) will each be analysed as continuous variates using a Mixed Model for Repeated Measures (MMRM) to assess any differences between the treatment arms. Each model will include the fixed categorical effects of treatment (i.e. prednisolone and placebo), time point (i.e. 6 weeks, 3 months and 6 months), whether a patient is anti-topoisomerase positive, baseline scores as well as the interactions of all fixed terms by time point. This will ensure the effects are permitted to vary for each of the three time points. The models will also be adjusted for any baseline characteristics found to be predictive of missing outcome values. A general unstructured covariance matrix will be used (six parameters). The models will be fitted using REstricted Maximum Likelihood (REML) and will employ Kenward and Roger degrees of freedom adjustment. The primary focus will be the contrast (adjusted mean difference) between treatment arms at 3 months.

There are a large number of secondary outcomes (listed in Table 2), and these will be analysed in an analogous manner to the co-primary outcomes. These analyses will be exploratory in nature each employing an unadjusted 2-tail 5% significance level for the trial arm comparison at 3 months.

Further exploratory analyses will compare the trial arms for the HAQ-DI, mRSS and other secondary outcomes at the other time points, that is, 6 weeks and 6 months, respectively. A single interim futility analysis is planned after approximately 30 participants have 3-month outcome data with early stopping only permitted for lack of benefit in both co-primary outcomes.

### Study setting

PRedSS is an initiative of the UK Scleroderma Study Group and will recruit from 16 centres in the United Kingdom. The initial plan was for 13 centres to recruit 72 patients over a 3-year period, but because of slow recruitment 3 new centres have been added.

## Results

Recruitment began at one centre in December 2017, with a second centre opening in March 2018. The first 12 centres opened by July 2019 and the 14th in January 2020: opening the final two centres is currently on hold with the Covid-19 pandemic. Opening centres took longer than planned but with over 12 centres open by the end of 2019 the study was recruiting as per the planned trajectory. To date, 25 patients have been recruited.

### Covid-19 impact

On 23/3/2020, the decision was taken to break the code for the 11 patients currently on trial treatment, as it was felt inappropriate for patients and their clinicians to remain blinded to which patients were receiving prednisolone treatment during the pandemic. This was against the background of the British Society for Rheumatology recommending that patients on both immunosuppressant therapy and prednisolone should be ‘social shielding’ as per National Health Service England guidance. Given that 10 of the 11 patients were on either mycophenolate mofetil or methotrexate, it was therefore considered untenable to maintain the double-blind. All 11 patients remained on trial on an open-label basis. Recruitment was suspended until deemed safe to re-open. Ethics approval has been obtained to convert PRedSS to a randomised open-label study, which is now a preferable approach given that ongoing concerns regarding Covid-19 would often make it difficult to maintain the double-blind.

## Discussion

PRedSS seeks to answer a key question for patients with early dcSSc and their clinicians – should corticosteroids be prescribed or not? If the answer is ‘yes’, then the pathway to impact will be immediate as prednisolone is a low-cost drug with which rheumatologists are very familiar.

Since PRedSS was opened, there have been no other studies reported specifically examining the use of corticosteroids in early dcSSc, although a study of high dose methylprednisolone in very early SSc (without skin involvement) (ClinicalTrials.gov Identifier: NCT03059979) is currently underway.^50^ In the recently reported North America study of autologous stem cell transplantation,^9^ only one of 34 patients randomised to the transplantation regimen, which included high dose corticosteroids (including six doses of intravenous methylprednisolone 1 mg/kg) developed scleroderma renal crisis. While this does not prove that corticosteroids are ‘safe’, it does lend some tentative support to our viewpoint that they may not be so nephrotoxic as to deprive patients with early dcSSc of the possibility of substantial symptomatic improvement, thus providing further rationale for PRedSS.

## References

[1] Rubio-Rivas M Royo C Simeón CP , et al. Mortality and survival in systemic sclerosis: systematic review and meta-analysis. Semin Arthritis Rheum 2014; 44(2): 208–219. 24931517 10.1016/j.semarthrit.2014.05.010

[2] Tyndall AJ Bannert B Vonk M , et al. Causes and risk factors for death in systemic sclerosis: a study from the EULAR Scleroderma Trials and Research (EUSTAR) database. Ann Rheum Dis 2010; 69(10): 1809–1815. 20551155 10.1136/ard.2009.114264

[3] Nihtyanova SI Sari A Harvey JC , et al. Using autoantibodies and cutaneous subset to develop outcome-based disease classification in systemic sclerosis. Arthritis Rheumatol 2020; 72(3): 465–476. 31682743 10.1002/art.41153

[4] Denton CP Hughes M Gak N , et al.; on behalf of the BSR and BHPR Standards, Guidelines and Audit Working Group. BSR and BHPR guideline for the treatment of systemic sclerosis. Rheumatology 2016; 55: 1906–1910. 27284161 10.1093/rheumatology/kew224

[5] Kowal-Bielecka O Fransen J Avouac J , et al. Update of the EULAR recommendations for the treatment of systemic sclerosis. Ann Rheum Dis 2017; 76: 1327–1339. 27941129 10.1136/annrheumdis-2016-209909

[6] Roumm AD Whiteside TL Medsger TA Jr , et al. Lymphocytes in the skin of patients with progressive systemic sclerosis. Arthritis Rheum 1984; 27(6): 645–653. 6375682 10.1002/art.1780270607

[7] Blagojevic J Legendre P Matucci-Cerinic M , et al. Is there today a place for corticosteroids in the treatment of scleroderma. Autoimmun Rev 2019; 18(12): 102403. 31639515 10.1016/j.autrev.2019.102403

[8] Van Laar JM Farge D Sont JK , et al. Autologous hematopoietic stem cell transplantation vs intravenous pulse cyclophosphamide in diffuse cutaneous systemic sclerosis. JAMA 2014; 311: 2490–2498. 25058083 10.1001/jama.2014.6368

[9] Sullivan KM Goldmuntz EA Keyes-Elstein L , et al. Myeloablative autologous stem-cell transplantation for severe scleroderma. N Engl J Med 2018; 378: 35–47. 29298160 10.1056/nejmoa1703327 PMC5846574

[10] Steen VD Medsger TA Jr. Case-control study of corticosteroids and other drugs that either precipitate or protect from the development of scleroderma renal crisis. Arthritis Rheum 1998; 41(9): 1613–1619. 9751093 10.1002/1529-0131(199809)41:9<1613::AID-ART11>3.0.CO;2-O

[11] DeMarco PJ Weisman MH Seibold JR , et al. Predictors and outcomes of scleroderma renal crisis: the high-dose versus low-dose D-penicillamine in early diffuse systemic sclerosis trial. Arthritis Rheum 2002; 46: 2983–2989. 12428241 10.1002/art.10589

[12] Guillevin L Bérezné A Seror R , et al. Scleroderma renal crisis: a retrospective multicentre study on 91 patients and 427 controls. Rheumatology (Oxford) 2012; 51(3): 460–467. 22087012 10.1093/rheumatology/ker271

[13] Nguyen B Mayes MD Arnett FC , et al. HLA-DRB1*0407 and *1304 are risk factors for scleroderma renal crisis. Arthritis Rheum 2011; 63(2): 530–534. 21280007 10.1002/art.30111 PMC3048905

[14] Hamaguchi Y Kodera M Matsushita T , et al. Clinical and immunologic predictors of scleroderma renal crisis in Japanese systemic sclerosis patients with anti-RNA polymerase III antibodies. Arthritis Rheumatol 2015; 67: 1045–1052. 25512203 10.1002/art.38994

[15] Herrick AL. Controversies on the use of steroids in systemic sclerosis. J Scleroderma Relat Disord 2017; 2: 84–91.

[16] Calguneri M Apras S Ozbalkan Z , et al. The efficacy of oral cyclophosphamide plus prednisolone in early diffuse systemic sclerosis. Clin Rheumatol 2003; 22(4–5): 289–294. 14579158 10.1007/s10067-003-0733-2

[17] Takehara K. Treatment of early diffuse cutaneous systemic sclerosis patients in Japan by low-dose corticosteroids for skin involvement. Clin Exp Rheumatol 2004; 22(Suppl. 33): S87–S89. 15344605

[18] Nadashkevich O Davis P Fritzler M , et al. A randomized unblinded trial of cyclophosphamide versus azathioprine in the treatment of systemic sclerosis. Clin Rheumatol 2006; 25(2): 205–212. 16228107 10.1007/s10067-005-1157-y

[19] Herrick AK Pan X Peytrignet S , et al. Treatment outcome in early diffuse cutaneous systemic sclerosis: the European Scleroderma Observational Study (ESOS). Ann Rheum Dis 2017; 76: 1207–1218. 28188239 10.1136/annrheumdis-2016-210503 PMC5530354

[20] Iudici M Fasano S Iacono D , et al. Prevalence and factors associated with glucocorticoids (GC) use in systemic sclerosis (SSc): a systematic review and meta-analysis of cohort studies and registries. Clin Rheumatol 2014; 33(2): 153–164. 24249145 10.1007/s10067-013-2422-0

[21] Steen VD Medsger TA Jr. The value of the health assessment questionnaire and special patient-generated scales to demonstrate change in systemic sclerosis patients over time. Arthritis Rheum 1997; 40(11): 1984–1991. 9365087 10.1002/art.1780401110

[22] Clements P Lachenbruch P Siebold J , et al. Inter- and intraobserver variability of total skin thickness score (modified Rodnan TSS) in systemic sclerosis. J Rheumatol 1995; 22(7): 1281–1285. 7562759

[23] Khanna D Furst DE Clements PJ , et al. Standardization of the modified Rodnan skin score for use in clinical trials of systemic sclerosis. J Scleroderma Relat Disord 2017; 2: 11–18. 28516167 10.5301/jsrd.5000231 PMC5431585

[24] Clements PJ Furst DE Wong WK , et al. High-dose versus low-dose D-penicillamine in early diffuse systemic sclerosis: analysis of a two-year, double-blind, randomized, controlled clinical trial. Arthritis Rheum 1999; 42(6): 1194–1203. 10366112 10.1002/1529-0131(199906)42:6<1194::AID-ANR16>3.0.CO;2-7

[25] Pope JE Bellamy N Seibold JR , et al. A randomized, controlled trial of methotrexate versus placebo in early diffuse scleroderma. Arthritis Rheum 2001; 44(6): 1351–1358. 11407694 10.1002/1529-0131(200106)44:6<1351::AID-ART227>3.0.CO;2-I

[26] Denton CP Merkel PA Furst DE , et al. Recombinant human anti-transforming growth factor β1 antibody therapy in systemic sclerosis: a multicentre, randomized, placebo-controlled Phase I/II trial of CAT-192. Arthritis Rheum 2007; 56: 323–333. 17195236 10.1002/art.22289

[27] Clements PJ Hurwitz EL Wong WK , et al. Skin thickness score as a predictor and correlate of outcome in systemic sclerosis. Arthritis Rheum 2000; 43(11): 2445–2454. 11083267 10.1002/1529-0131(200011)43:11<2445::AID-ANR11>3.0.CO;2-Q

[28] Furst DE. Outcome measures in rheumatologic clinical trials and systemic sclerosis. Rheumatology (Oxford) 2008; 47(Suppl. 5): v29–v30. 18784136 10.1093/rheumatology/ken269

[29] Silman A Akesson A Newman J , et al. Assessment of functional ability in patients with scleroderma: a proposed new disability assessment instrument. J Rheumatol 1998; 25(1): 79–83. 9458207

[30] Man A Correa JK Ziemek J , et al. Development and validation of a patient-reported outcome instrument for skin involvement in patients with systemic sclerosis. Ann Rheum Dis 2017; 76(8): 1374–1380. 28213563 10.1136/annrheumdis-2016-210534

[31] Elman S Hynan LS Gabriel V , et al. The 5-D itch scale: a new measure of pruritus. Br J Dermatol 2010; 162(3): 587–593. 19995367 10.1111/j.1365-2133.2009.09586.x PMC2875190

[32] El-Baalbaki G Razykov I Hudson M , et al. Association of pruritus with quality of life and disability in systemic sclerosis. Arthritis Care Res (Hoboken) 2010; 62(10): 1489–1495. 20506531 10.1002/acr.20257

[33] Rannou F Poiraudeau S Berezne A , et al. Assessing disability and quality of life in systemic sclerosis: construct validities of the Cochin Hand Function Scale, Health Assessment Questionnaire (HAQ), Systemic Sclerosis HAQ, and Medical Outcomes Study 36-Item Short Form Health Survey. Arthritis Rheum 2007; 57: 94–102. 17266096 10.1002/art.22468

[34] Peytrignet S Denton CP Lunt M , et al. Disability, fatigue, pain and their associates in early diffuse cutaneous systemic sclerosis: the European Scleroderma Observational Study. Rheumatology 2018; 57: 370–381. 29207002 10.1093/rheumatology/kex410 PMC5850714

[35] Webster K Cella D Yost K. The Functional Assessment of Chronic Illness Therapy (FACIT) Measurement System: properties, applications, and interpretation. Health Qual Life Outcomes 2003; 1: 79. 14678568 10.1186/1477-7525-1-79 PMC317391

[36] Harel D Thombs BD Hudson M , et al. Measuring fatigue in SSc: a comparison of the Short Form-36 Vitality subscale and Functional Assessment of Chronic Illness Therapy–Fatigue scale. Rheumatology (Oxford) 2012; 51(12): 2177–2185. 22923749 10.1093/rheumatology/kes206

[37] Thombs BD Hudson M Bassel M , et al. Sociodemographic, disease, and symptom correlates of fatigue in systemic sclerosis: evidence from a sample of 659 Canadian Scleroderma Research Group Registry patients. Arthritis Care Res 2009; 61: 966–973. 10.1002/art.24614 19565539

[38] Zigmond AS Snaith RP. The hospital anxiety and depression scale. Acta Psychiat Scand 1983; 67: 361–370. 6880820 10.1111/j.1600-0447.1983.tb09716.x

[39] Del Rosso A Mikhaylova S Baccini M , et al. In systemic sclerosis, anxiety and depression assessed by Hospital Anxiety Depression Scale are independently associated with disability and psychological factors. Biomed Res Int 2013;2013:507493. 23984376 10.1155/2013/507493 PMC3745942

[40] Nguyen C Ranque B Baubet T , et al. Clinical, functional and health-related quality of life correlates of clinically significant symptoms of anxiety and depression in patients with systemic sclerosis: a cross-sectional survey. PLoS ONE 2014; 9(2): e90484. 24587375 10.1371/journal.pone.0090484 PMC3938731

[41] Nicassio PM Wallston KA Callahan LF , et al. The measurement of helplessness in rheumatoid arthritis. J Rheumatol 1985; 12(3): 462–467. 4045844

[42] DeVellis RF Callahan LF. A brief measure of helplessness in rheumatic disease: the helplessness subscale of the Rheumatology Attitudes Index. J Rheumatol 1993; 20(5): 866–869. 8336314

[43] Ware JE . The MOS 36-item short-form health survey (SF-36). 1. Conceptual framework and item selection. Med Care 1992; 30: 473–483. 1593914

[44] EuroQol Group. EuroQol – a new facility for the measurement of health-related quality of life. Health Policy 1990; 16(3): 199–208. 10109801 10.1016/0168-8510(90)90421-9

[45] Clements PJ Allanore Y Khanna D , et al. Arthritis in systemic sclerosis: systematic review of the literature and suggestions for the performance of future clinical trials in systemic sclerosis arthritis. Semin Arthritis Rheum 2012; 41(6): 801–814. 22177105 10.1016/j.semarthrit.2011.10.003

[46] Khanna D Berrocal VJ Giannini EH , et al. The American College of Rheumatology provisional composite response index for clinical trials in early diffuse cutaneous systemic sclerosis. Arthritis Care Res 2016; 68: 167–178. 10.1002/acr.22804 PMC4818571 26806474

[47] Borm GF Fransen J Lemmens WA. A simple sample size formula for analysis of covariance in randomized clinical trials. J Clin Epidemiol 2007; 60(12): 1234–1238. 17998077 10.1016/j.jclinepi.2007.02.006

[48] Herrick AL Lunt M Whidby N , et al. Observational study of treatment outcome in early diffuse cutaneous systemic sclerosis. J Rheumatol 2010; 37: 116–124. 19955050 10.3899/jrheum.090668

[49] Khanna D Furst DE Hays RD , et al. Minimally important difference in diffuse systemic sclerosis: results from the D-penicillamine study. Ann Rheum Dis 2006; 65(10): 1325–1329. 16540546 10.1136/ard.2005.050187 PMC1798331

[50] Van den Hombergh WM Kersten BE Knaapen-Hans HK , et al. Hit hard and early: analysing the effects of high-dose methylprednisolone on nailfold capillary changes and biomarkers in very early systemic sclerosis: study protocol for a 12-week randomised controlled trial. Trials 2018; 19: 449. 30134971 10.1186/s13063-018-2798-x PMC6104002

